# Butyrate-Dependent Conformational Switch Promotes Nuclear Translocation of the Mustard Allergen Sin a 1 in Human Gut Epithelial Cells

**DOI:** 10.1021/acs.jafc.6c01248

**Published:** 2026-06-11

**Authors:** Rubén G. Gordo, Jorge Parrón-Ballesteros, Nieves Olmo, Mayte Villalba, Eva Batanero, Javier Turnay

**Affiliations:** Department of Biochemistry and Molecular Biology, Faculty of Chemistry, Complutense University of Madrid, 28040 Madrid, Spain

**Keywords:** 2S albumins, butyrate, Caco-2 cells, food allergy, Sinapis alba, nuclear transport, Sin a 1 allergen

## Abstract

Sin a 1, the major allergen from mustard seeds, is a highly stable 2S albumin capable of reaching the intestinal epithelium in its intact form. In this study, we investigated whether butyrate, a microbiota-derived short-chain fatty acid, directly interacts with Sin a 1 and modulates its cellular behavior in Caco-2 cells. Circular dichroism analyses revealed that short-chain fatty acids induce conformational changes in Sin a 1. However, fluorescence spectroscopy showed that only butyrate modified the tertiary structure of the light chain, exposing a putative nuclear localization signal and thereby promoting the importin-dependent nuclear translocation of the allergen. In the absence of butyrate, Sin a 1 remained predominantly in the cytoplasm. In addition, butyrate altered NFKB1 transcriptional responses in Sin a 1-treated cells. Our findings reveal an effect of butyrate on the intracellular trafficking of allergens that had not previously been described, suggesting a novel mechanism by which microbiota-derived metabolites may influence food allergenicity.

## Graphical abstract

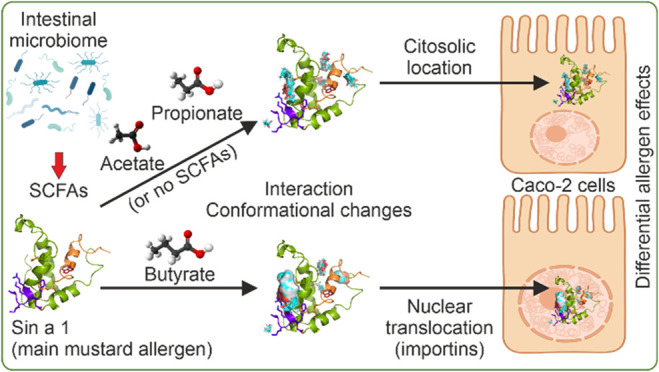

## 1 Introduction

Dietary fiber metabolized by a healthy gut microbiota has been shown to exert beneficial effects on the immune environment not only in the gut but also in distant organs (i.e., lung or skin), protecting against the development of food, respiratory, or contact allergies.^1,2^ Among these metabolites, fiber-derived short-chain fatty acids (SCFAs) have been shown to possess potent anti-inflammatory properties and improve epithelial integrity.^1,3^ The connection among the gut microbiome, allergy, and the protective influence of microbiome-produced butyrate underscores the need to understand the intricate interplay among diet, microbiome, and human health.

Butyrate plays a key role in this microbial narrative;^4,5^ it induces changes that enhance the integrity of the intestinal barrier, a key factor in protection against allergic diseases.^3,6^ The interaction of butyrate with specific receptors on intestinal and immune cells has been described, triggering desensitization of stimuli and inhibiting inflammatory responses.^7,8^ It orchestrates the differentiation of *T*_reg_ cells in the colon,^9,10^ leading to STAT3 promoter activation on naïve T cells.^11,12^ The impact of butyrate extends beyond metabolism, as it has a role in the epigenetic regulation of the human epithelium through its ability to inhibit histone deacetylases.^13–15^ This drives the activation of STAT3 and other key factors in immune cell development,^16,17^ contributing to the maintenance of epithelial barrier integrity.^3,18^ Butyrate therefore plays a pivotal role in regulating immune responses, reinforcing the epithelial barrier structure, and maintaining homeostasis in mucous membranes.^13,18,19^ Currently, research on butyrate focuses on its interaction with and direct effect on cells, leading to a tolerogenic response to potential allergens.^20–22^ This opens new therapeutic and preventive strategies for the management of allergies, establishing the gut microbiota as a promising target for innovative treatments.

The objective of this study was to explore the specific role of butyrate in controlling epithelial responses to allergenic dietary elements and the pathways involved in this process. Food allergies affect around 8% of the global population, leading to reduced quality of life, increased mortality, and substantial healthcare costs. Storage proteins such as 2S albumins, which are found in fruit seeds and nuts, are associated with numerous food allergies that can cause severe symptoms.^23^ In addition to being ubiquitous components of many foods, these proteins are also present in a wide variety of processed and ready-to-eat products, often in trace amounts. Together with their growing popularity in human diets due to their nutritional benefits, this widespread presence has contributed to increased consumption and, consequently, to the rising prevalence of associated allergies.

Consequently, we have selected a member of this food allergenic family as a model, specifically Sin a 1, the major allergen from yellow mustard (*Sinapis alba*) seeds. As with any allergen, Sin a 1 can induce, in atopic patients, a Th2-mediated immune response consisting of sensitization and effector phases.^3^ This 2S albumin demonstrates exceptional stability during both gastric and intestinal digestion, along with a high thermostability.^24–26^ As a result, it is an excellent model allergen for studying epithelial responses in the intestine as it reaches this location intact. Sin a 1 isoforms are basic proteins (pI 8–9) that comprise two polypeptide chains, referred to as light and heavy (39 and 91 residues, respectively), that are held together and stabilized by disulfide bridges.^24,27^ Following processing and removal of the propeptide (15 residues), it attains a molecular mass of 14.6 kDa.^24,28,29^

Apart from the above-described potential beneficial effects of butyrate regarding intestinal epithelial cells, we wanted to know whether there was a direct interaction of this SCFA with Sin a 1 and if this interaction altered the internalization of the allergen or its subcellular localization, effects that could also be involved in decreasing the allergic response to the main allergen present in mustard seeds.

To replicate the intestinal epithelium and investigate its response to the selected model allergen, we have used the well-established and nondifferentiated human colorectal adenocarcinoma cell line Caco-2.^30,31^ The available literature indicates that food allergies most commonly develop during early childhood, likely due to an immature or disrupted intestinal epithelium.^32–34^ In addition, the “epithelial barrier theory” states that the disruption or impairment of the barrier also facilitates sensitization to allergens.^35,36^ For this reason, we employed undifferentiated Caco-2 cells to emulate such immature and/or impaired epithelium in these analyses.

## 2 Materials and Methods

### 2.1 Culture Conditions and Treatments

Human colorectal adenocarcinoma Caco-2 cells (ATCC HTB-38) between passages 8 and 15 were routinely seeded at 3 × 10^3^ cells/cm^2^, incubated at 37 °C in a humidified atmosphere containing 5% CO_2_ in DMEM-High Glucose culture medium (Cytiva, Hospitalet de Llobregat, Spain) supplemented with 10% fetal bovine serum (Corning, Madrid, Spain), 2 mM glutamine, 1% nonessential amino acids and 100 U/mL penicillin and 100 μg/mL streptomycin on 75 cm^2^ tissue culture flasks (Corning). For confocal laser microscopy, cells were grown on round glass coverslips in 24-well culture plates (Corning).

SCFAs (butyrate, propionate, and acetate) (Sigma-Aldrich, Madrid, Spain) and allergen treatments were carried out on Caco-2 cell cultures (20,000 cells/cm^2^) 3 days postconfluence; at this stage, the cells are still in a nondifferentiated state, which may mimic an impaired epithelium that is more susceptible to allergic sensitization. Stock solutions of SCFAs (40 mM) and Sin a 1 (1 mg/mL) were prepared in PBS. Cells were treated (unless otherwise stated) for 24 h in the presence or absence of 1 mM SCFAs in complete culture medium, followed by an additional 24 h in the presence or absence of SCFAs and allergens at 40 μg/mL (∼3 μM for 2S albumins and 4.3 μM for Pru p 3). The butyrate concentration was selected based on physiological concentrations found in the small intestine of this SCFA, and after checking that incubation conditions did not induce significant changes in cell viability (MTT assay) nor in the differentiation degree (ALP assay) using protocols previously described by our group.^37^

### 2.2 Allergen Purification and Labeling

Mustard seed extracts were prepared in our laboratory as previously described^38^ with minor modifications (see Supporting Information for details). Sin a 1 was purified from the lyophilized protein extract after resuspension in 20 mM ammonium bicarbonate, pH 6.5, filtration through 0.20 μm (SCFA-PF; Corning), loading onto a MonoS HR 5/5 column (Cytiva) in an Äkta Purifier FPLC system (Amershan Biosciences, Hospitalet de Llobregat, Spain) and elution with a NaCl gradient (0–1 M). Fractions were analyzed by sodium dodecyl sulfate-polyacrylamide gel electrophoresis (SDS-PAGE) and Western blot to select those containing Sin a 1, which were desalted and pooled together (Figure S1). The average purification yield was approximately 1 mg of pure Sin a 1 per 10 g of lyophilized delipidated seed extracts. Seed storage 2S albumins Pis v 1 (from pistachio) and Ana o 3 (from cashew nuts), and nsLTP Pru p 3 (from peach skin) were purified according to published protocols.^38,39^

Labeling of allergens with Pacific-Blue (PB) or with Alexa Fluor 488 (AF^488^) was carried out with Pacific-Blue Protein Labeling Kit (Invitrogen, Alcobendas, Spain) or Ester-NHS Alexa Fluor 488 Kit (Fisher Scientific). Free dyes were removed using PD-10 columns with PBS as elution buffer.

### 2.3 Analytical Procedures

SDS-PAGE was carried out in 17% polyacrylamide gels under both nonreducing and reducing [5% β-mercaptoethanol (v/v)] conditions, stained with Coomassie Blue R-250 or, alternatively, transferred to 0.45 μm Hybond nitrocellulose membranes using a semidry system (Amersham Biosciences) for Western blot.^40^ Polyclonal antibodies against Sin a 1 (provided by Dr. Pastor-Vargas, Complutense University of Madrid; 1:2000) or human histone H3 (9715, 1:250, Cell Signaling, Biosciences), and monoclonal antibodies against human vinculin (hVIN-1, 1:2000, Sigma-Aldrich) were used, followed by HRP-conjugated goat antirabbit (31437X, 1:5000, Invitrogen, Alcobendas, Spain) or antimouse (31430, 1:5000, Thermo Scientific) IgG. Band development was achieved using ECL Western Blotting Substrate (Thermo Scientific, Alcobendas, Spain), and images were captured at different exposure times in a ChemiDoc XRS+ system (Bio-Rad, Alcobendas, Spain).

Allergen concentrations were determined from UV–visible spectra using molar extinction coefficients at 280 nm [ε (Sin a 1) = 7490 M^–1^·cm^–1^; ε (Pis v 1) = 11,960 M^–1^·cm^–1^; ε (Ana o 3) = 6460 M^–1^·cm^–1^; ε (Pru p 3) = 3480 M^–1^·cm^–1^].

### 2.4 Confocal Laser Scanning Microscopy

Subcellular localization of Sin a 1 was first analyzed by confocal laser scanning microscopy (CLSM) using AF^488^-labeled allergen under the experimental conditions described above in the presence or absence of 1 mM SCFAs. Parallel experiments were carried out with the other labeled allergens. Following the completion of the treatment and the removal of the culture medium, cells were fixed with 4% paraformaldehyde (w/v) for 10 min, washed three times with 0.05% (v/v) PBS-Tween 20 for 5 min, permeabilized with 0.5% PBS-Triton X-100 for 10 min, washed again, and blocked with 3% BSA (w/v) in PBS-Tween 20 for 1 h. After further washing, preparations were stained with different combinations of primary monoclonal antibodies [anti-Zonula Occludens-1 (ZO-1, BLR092G, 1:1,000) and anti-14-3-3ζ, (818515, 1:2,500), both from Novus Biologicals, Móstoles, Spain] followed by secondary goat antimouse-Alexa^647^, or fluorescent probes: LysoTraker Red (75 nM) for lysosome/acidic compartments visualization, DAPI (4,6-diamidino-2-phenylindole; included in Prolong Gold) for nuclei, and phosphatidyl­(N-sulphorhodamine B sulphonyl)­ethanolamine (N-Rh-PE; 1 μM) for multilamellar vesicles (all from Thermo Scientific). Finally, cell-containing coverslips were mounted on slides using antifading fluorescence mounting medium (Dako Omnis, Las Rozas, Spain). Samples were analyzed using an Olympus FluoView-1200 microscope from the UCM Fluorescence Microscopy Unit and processed using ImageJ software. Twenty different scans with Z-stacks captured at 10 μm intervals were analyzed for each experimental condition.

The raw binary CLSM images were processed using the Coloc2 plugin from ImageJ (Fiji, NIH) to determine the colocalization of allergens with cell elements. Color channels were separated, and each layer of Z-stacks was analyzed, using the complete image, without adding ROIs. Thresholds were applied to eliminate background noise, running the method only on pixels with real color information (noise elimination). The colocalization data were expressed as Manders’ Overlap Coefficients,^41^ where a value of 1 indicates 100% colocalization of the analyzed fluorescent probes.

### 2.5 Cytoplasmic/Nuclear Extraction and Sin a 1 Localization

Nuclear localization of Sin a 1 in the presence of butyrate was also studied after incubation of Caco-2 cells, as described above, but using PB-labeled Sin a 1. After removal of the culture medium and washing the monolayer with PBS, cells were lysed in 10 mM Tris, pH 7.4, containing 140 mM NaCl and 1% Triton X-100, followed by separation of nuclear and cytoplasmic extracts using the NE-PER Nuclear and Cytoplasmic Extraction Kit (Thermo Scientific). The protein concentration in the extracts was determined using the Bradford assay, and equivalent amounts of protein were analyzed by SDS-PAGE and Western blot, using antivinculin (cytoplasm) and antihistone H3 (nucleus) antibodies to check the specificity of the separation (Figure S2). Aliquots of the nuclear and cytoplasmic extracts were transferred to 96 well plates, and fluorescence emission was measured in a Fluostar Optima (BMG Labtech, Ortenberg, Germany) using excitation and emission wavelengths of 405 and 490 nm, respectively, and normalized first to protein concentration and subsequently to their corresponding controls without labeled Sin a 1 (since control samples exhibited basal fluorescence; *F*_490_ = 100%).

### 2.6 Nuclear Translocation Inhibition Assay

Nuclear translocation of Sin a 1 in the presence of butyrate was analyzed by inhibition of the main nuclear translocation route, the α/β-importin-mediated transport, by coincubation of the allergen (after a 24 h preincubation with or without 1 mM butyrate) with 5 μM ivermectin (α/β-importin inhibitor) or 40 μM importazole (β-importin inhibitor) (both from Merck, Madrid, Spain) for 24 h in the presence or absence of 1 mM butyrate. Concentrations of the inhibitors were selected based on previous studies demonstrating efficient inhibition of α/β-importin-mediated nuclear transport in cultured cells.^42,43^ Stock solutions of both inhibitors were prepared in DMSO and were further diluted in complete culture medium; DMSO concentration was maintained below 0.5% and was added to the corresponding controls.

### 2.7 Mimicking Butyrate HDAC Inhibitory Activity

To check whether the inhibition of HDAC activity by butyrate participated in the nuclear transport of Sin a 1, butyrate was replaced by different concentrations of trichostatin-A (TSA; stock solutions in DMSO), a potent HDAC inhibitor. Applied conditions were as previously determined in our group.^4,44^ Since TSA is a more potent HDAC inhibitor than butyrate, instead of 24 h preincubation with the inhibitor, cells were directly incubated for 24 h in the presence of Sin a 1-AF^488^, without or with TSA (0.125, 0.25, and 0.5 μM).

### 2.8 Quantitative Real-Time PCR (qRT-PCR)

Total RNA from cultured cells was isolated using TRIzol Plus RNA Purification kit (Invitrogen) following the manufacturer’s instructions. RNA concentration and purity were assessed using a NanoDrop One spectrophotometer (Thermo Scientific) and a 4150 TapeStation System (Agilent, Las Rozas, Spain). Complementary DNA (cDNA) was synthesized from 500 ng of total RNA via reverse transcription using the PrimeScript RT Master Mix (Takara Bio Inc., Saint-Germain-en-Laye, France). qPCR was performed using PowerUp SYBR Green Master Mix reagents (Applied Biosystems, Alcobendas, Spain) on a QuantStudio 7 system (Applied Biosystems) with 1–10 dilutions of cDNA as the template and specific primers at 300 nM final concentration. The assay was performed in 384-well format, using the following thermal cycling protocol: 95 °C/10 min, followed by 40 cycles with 95 °C/15 s, and 60 °C/1 min, at the Genomics facilities of the Complutense University of Madrid. Primer sequences are provided in Table S1.

### 2.9 Spectroscopical Characterization of the Allergen

Far-UV circular dichroism (CD) spectra were monitored between 195 and 260 nm at 20 °C in a Jasco J-715 spectropolarimeter equipped with a Neslab RTE-111 bath using 0.05 cm path length thermostatized cuvettes and with a protein concentration of 0.3 mg/mL (21 μM) in 10 mM HEPES, 0.1 M NaCl, pH 6.5. Allergen spectra in the presence of SCFAs were obtained at different protein-to-SCFA molar ratios (1:1, 1:10 and 1:50). All spectra were averaged over eight scans and corrected by subtracting buffer contribution from reference spectra in the absence of protein; units were expressed as mean residue-weighed molar ellipticities (θ_MRW_). Prediction of the secondary structure from the far-UV CD spectra was performed using the CDNN^45^ and CCA algorithm.^46^

Fluorescence emission spectra were recorded in an SLM Aminco 8000C spectrofluorimeter at 20 °C. Global and Trp emissions were separately measured with excitation wavelengths of 275 and 295 nm, respectively, using a 0.4 cm excitation path length and 1.0 cm emission path length cuvette, and 0.1 mg/mL (7 μM) protein concentration. Spectra were monitored between 265 and 420 nm for global emission or 285 and 420 nm for Trp emission. The emission spectra from the tyrosine residue (Y^117^) were determined after subtracting the contribution of the single Trp residue (W^26^) to the global emission spectra (λ_ex_ = 275 nm). This contribution was obtained by correction of the Trp spectra (λ_ex_ = 295 nm) using a factor determined from the ratio between both spectra (*F*_ex=275_/*F*_ex=295_) in the range from 370 to 400 nm.

The effect of SCFA binding to Sin a 1 in the emission spectra was measured after the sequential addition of stock solutions of the fatty acids. Baseline spectra in the absence of proteins were recorded at all SCFA concentrations. Care was taken to avoid the inner filter effect, and solutions always presented UV absorption below 0.05 at both excitation wavelengths.

### 2.10 Data Analysis and *In Silico* Processing

ImageJ 1.54f (Wayne Rasband, NIH) software was employed for CLSM image visualization and analysis. CD data were processed using Spectra Manager software (Jasco), and secondary structure prediction was obtained using the CDNN and CCA algorithm. SigmaPlot 16 (Systat Software, Inc.) was used for graphic representation of data and statistical analyses. Mentioned analyses were performed applying one-, two-, or three-way ANOVA depending on the desired analysis and the number of variables studied. Statistical significance was set at *p* < 0.05.

Three-dimensional (3D) structural models were obtained using the Swiss-Model^47^ and the AlphaFold 3 ^48^ servers. Docking prediction of SCFAs on Sin a 1 was carried out with the SwissDock server,^49,50^ using the Sin a 1 Swiss-Model or the AlphaFold 3 3D-structures. Protein structure images were generated with The PyMol Molecular Graphics System, Version 3.1 (Schrödinger, LLC.), which was also used to calculate the solvent accessible surface areas of Trp and Tyr residues.

## 3 Results

### 3.1 Sin a 1 Translocates to the Nucleus of Caco-2 Cells in the Presence of Butyrate

Fluorescent staining was employed to visualize the intracellular localization in nondifferentiated Caco-2 cells not only of Sin a 1-AF^488^, but also of the allergenic-response mediator protein 14-3-3ζ,^51^ tight junction protein ZO-1, nuclei, multilamellar vesicles (MLVs), and acidic compartments. Figure 1 shows representative images of control cells stained with ZO-1, LysoTracker red, and DAPI (Figure 1A), in addition to cells treated only with butyrate (Figure 1B), with Sin a 1 (Figure 1C), or with Sin a 1 plus SCFAs (Figure 1D–F). Butyrate-treated cells without Sin a 1 show staining patterns comparable to those of control cells, although the cells appeared slightly smaller. We analyzed the colocalization of Sin a 1-AF^488^ with the above-mentioned proteins or cellular compartments on each image throughout all Z-stack sections, obtaining an overall colocalization value of the complete image. According to these analyses, under control conditions, Sin a 1 is mainly located in the cytoplasm of certain cells (Sin a 1-DAPI Manders’ coefficient: 0.38). Furthermore, no colocalization was observed of Sin a 1-AF^488^ with lysosomes or multilamellar vesicles, suggesting that these compartments are unlikely to participate in allergen processing (Table 1). However, Sin a 1 is internalized by Caco-2 cells and, once in the cytoplasm, the allergen interacts with allergy-related mediator proteins, as it colocalizes with the allergenic-response mediator 14-3-3ζ protein with a Manders’ coefficient of 0.93 (Table 1), suggesting that the cytoplasmic location of Sin a 1 may be related to its allergy-inducing effects both *in vitro* and in patients.^28,52^

**Figure 1:**
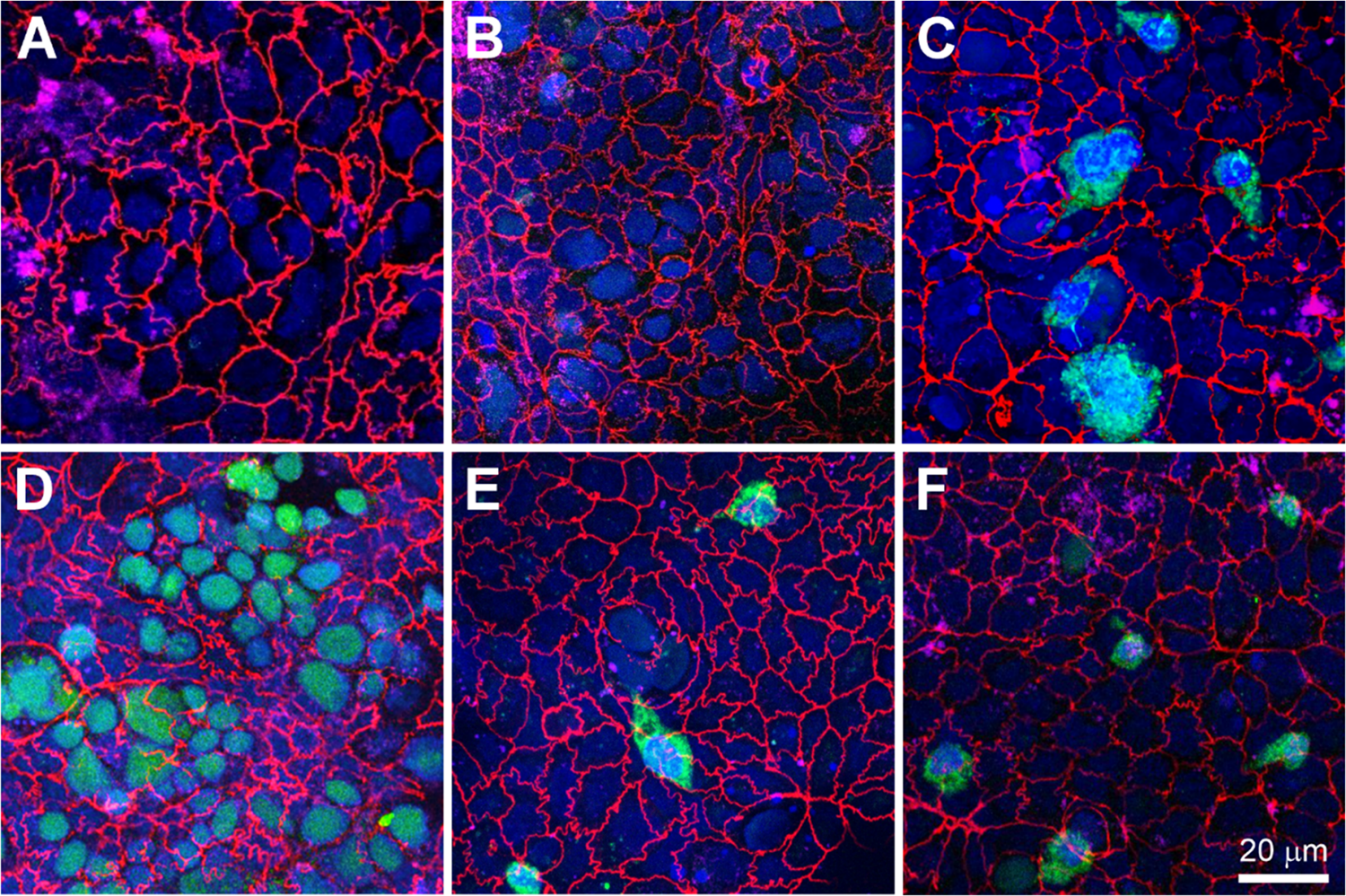
Subcellular localization of Sin a 1 in the absence or presence of SCFAs in Caco-2 cells analyzed by CLSM. Control cells without Sin a 1 and without (A) or with 1 mM butyrate (B); (C) cells incubated with 40 μg/mL Sin a 1-AF^488^ (green) in the absence of SCFAs, or in the presence of 1 mM butyrate (D), 1 mM acetate (E), or 1 mM propionate (F). Incubations were carried out for 24 h in the absence or presence of SCFAs, followed by an additional 24 h incubation after the addition of Sin a 1-AF^488^. Nuclei were stained with DAPI (blue), tight junctions with a monoclonal antibody anti-Zonula Occludens-1 (red), and acidic compartments with LysoTracker Red (magenta).

**Table 1:** Colocalization of Sin a 1 with Different Markers^a^

	DAPI	14-3-3ζ	LysoTracker Red	N-Rh-PE
control	0.38 ± 0.11	0.93 ± 0.04	0.24 ± 0.16	0.11 ± 0.11
butyrate (1 mM)	0.93 ± 0.07	0.89 ± 0.09	0.30 ± 0.14	0.30 ± 0.03

a Data represent mean Manders’ colocalization coefficients of Sin a 1 with the corresponding markers (±SD) obtained from the analysis of 20 CLSM images from 3 independent experiments, using 10 μm Z-stacks.

Interestingly, Caco-2 cells incubated together with Sin a 1-AF^488^ and 1 mM butyrate for 24 h, exhibited a nuclear localization of Sin a 1, with little to no allergen detected in the cytoplasm (Figure 1D). Quantification of the colocalization of Sin a 1 with DAPI and with 14-3-3ζ protein yielded Manders’ coefficients of 0.93 and 0.89, respectively (Table 1). To check whether nuclear import of Sin a 1 was specifically induced by butyrate or could be dependent on other microbiome-derived SCFAs, the experiments were repeated under identical conditions with 1 mM acetate (Figure 1E) or propionate (Figure 1F). However, neither acetate nor propionate induced nuclear translocation of Sin a 1 at 1 mM (Manders’ coefficients 0.40 and 0.43, respectively) or even at higher concentrations up to 10 mM (data not shown).

To confirm the butyrate-induced nuclear translocation of Sin a 1, we carried out a nuclear/cytoplasm fractionation of Caco-2 cells treated with Sin a 1 labeled with Pacific-Blue in the absence or presence of 1 mM butyrate. We analyzed the relative fluorescence emission at 490 nm (λ_ex_ = 405 nm) of nuclear and cytoplasmic extracts normalized to total protein content. Sin a 1-PB fluorescence in the cytoplasm was masked by the intrinsic background fluorescence of the samples, and no differences were found between controls and samples with Sin a 1. However, when the nuclear extract was analyzed (Figure 2), a significant increase in fluorescence emission at 490 nm was detected in butyrate/Sin a 1-treated cells compared to those incubated with Sin a 1 alone, confirming the nuclear localization of the allergen in the presence of butyrate.

**Figure 2:**
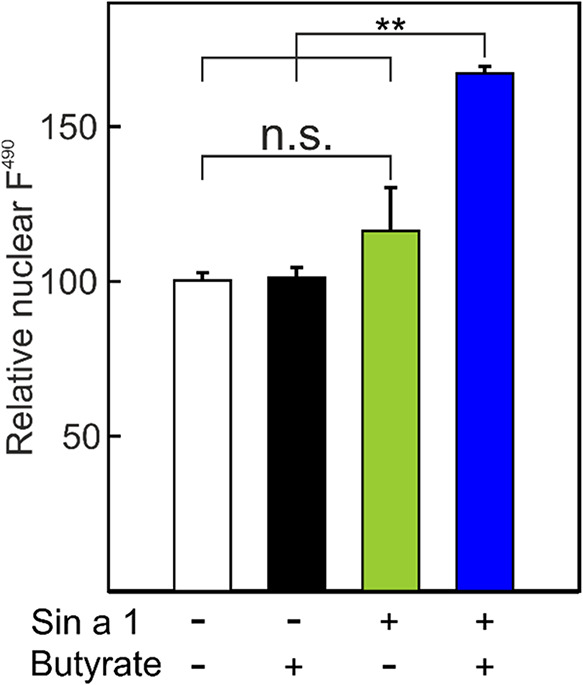
Sin a 1-PB fluorescence in nuclear extracts from Caco-2 cells. Relative fluorescence emission at 490 nm from nuclear extracts of Caco-2 cells incubated with Sin a 1-PB in the absence or presence of 1 mM butyrate under the same conditions as in Figure 1. Experiments were repeated at least twice with quadruplicate samples. Data represent mean fluorescence values normalized to protein content ± SD. Untreated control cells showed intrinsic fluorescence that was considered as 100%. (**) *p* < 0.001; (n.s.) not significant.

In addition, we wanted to know whether nuclear translocation induced by butyrate was a general characteristic of allergenic 2S albumins or even of other prolamins. For this purpose, we carried out parallel experiments with the 2S albumins Pis v 1 and Ana o 3, and the nsLTP Pru p 3 at the same concentration used for Sin a 1 (40 μg/mL). As observed in Figure 3 (Manders’ coefficients in Table S2), these three allergens remained in the cytoplasm in the presence of 1 mM butyrate (as well as with propionate or acetate; not shown).

**Figure 3:**
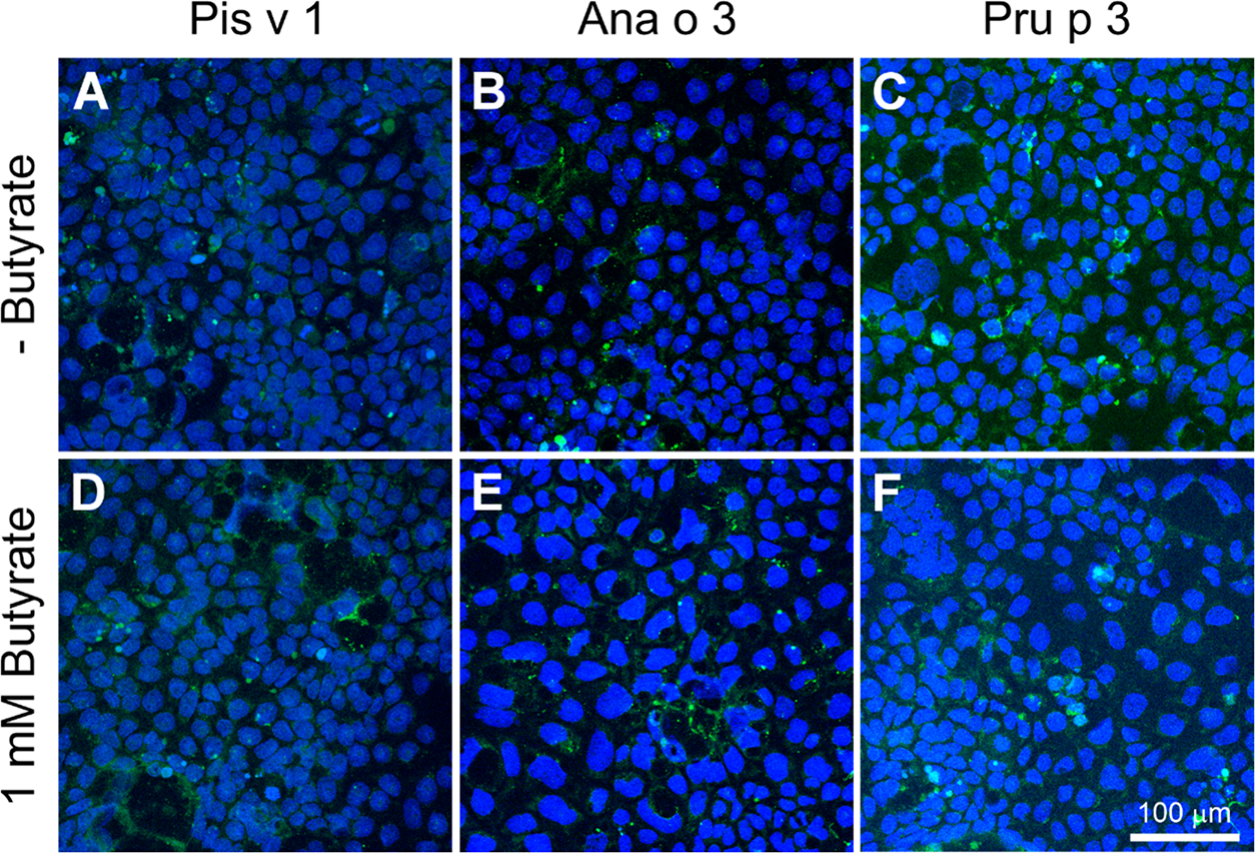
Subcellular localization of labeled allergens in the absence or presence of butyrate in Caco-2 cells analyzed by CLSM. Caco-2 cells were incubated without butyrate (A, B, C) or with 1 mM butyrate (D, E, F) in the presence of 40 μg/mL of the corresponding AF^488^-labeled allergen (green). Incubations were carried out for 24 h in the absence or presence of SCFAs, followed by an additional 24 h incubation after the addition of AF^488^-labeled allergens. Nuclei were stained with DAPI (blue).

### 3.2 Mechanism of Sin a 1 Nuclear Translocation Induced by Butyrate

To determine whether the nuclear translocation of Sin a 1 in the presence of butyrate was dependent on importins, we incubated Caco-2 cells as previously described, with two different importin inhibitors: importazole (β-importin inhibitor) and ivermectin (α/β-importins inhibitor). CLSM images (Figure 4) revealed that 5 μM ivermectin or 40 μM importazole, even after only 1 h incubation, inhibited Sin a 1 translocation to the nucleus in butyrate-treated Caco-2 cells, showing that nuclear import of Sin a 1 was mediated by α/β-importins. Manders’ coefficients of Sin a 1 and DAPI colocalization on butyrate-treated cells in the presence of ivermectin or importazole decreased from 0.93 (in the absence of inhibitors) to 0.30 and 0.32, respectively, which were similar to those from butyrate-untreated cells in the presence of inhibitors (0.33 and 0.20, respectively) (Figure 4 and Table 2). To our knowledge, this is the first report describing an allergen transported into the mammalian cell nucleus via α/β-importins.

**Figure 4:**
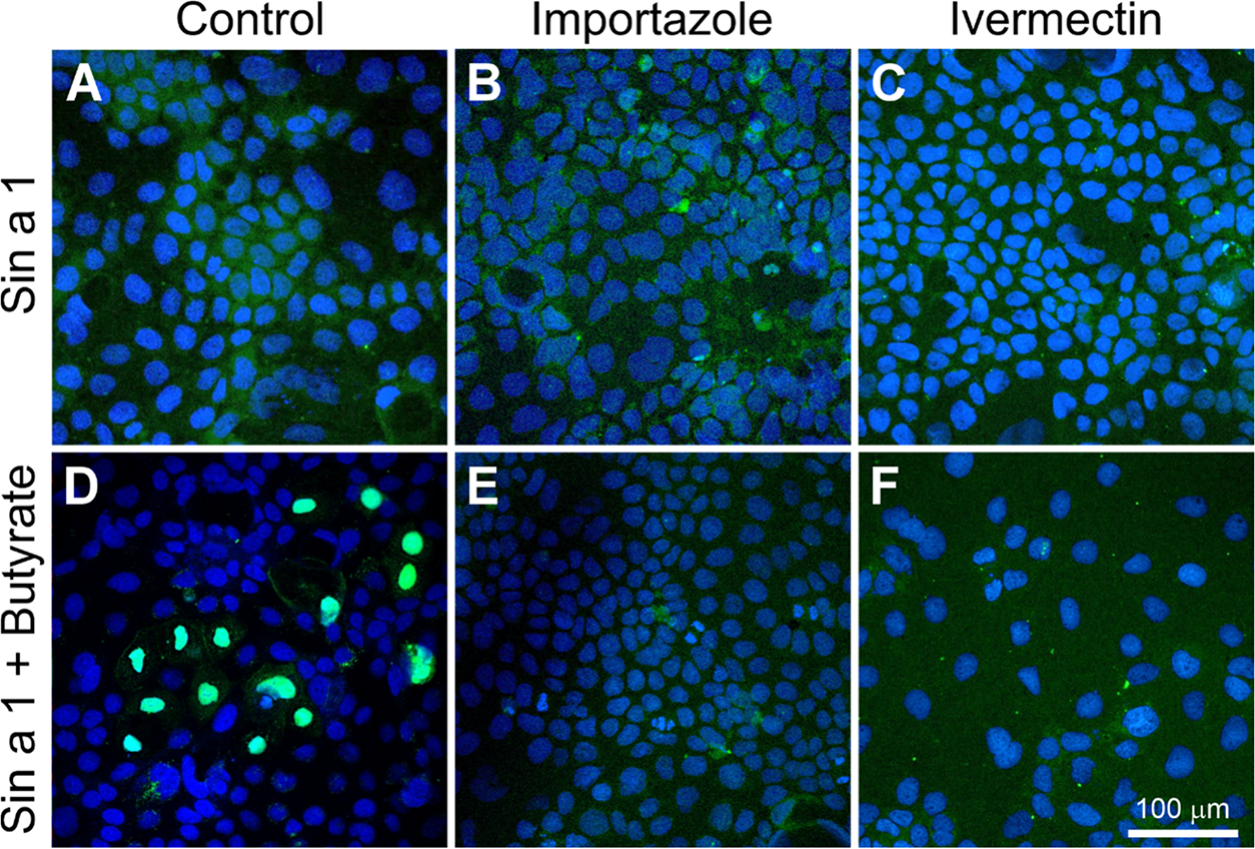
Inhibition of α/β-importins blocks nuclear translocation of Sin a 1 in the presence of butyrate. Caco-2 cells were treated for 24 h with or without 1 mM butyrate (control) followed by an additional 24 h of coincubation with 40 μg/mL Sin a 1-AF^488^ (green) and without (A) or with 1 mM butyrate (D). These conditions were combined with treatments with α/β-importin inhibitors: 40 μM importazole (B, E) or 5 μM ivermectin (C, F). Nuclei were stained with DAPI (blue).

**Table 2:** Nuclear Localization of Sin a 1 under Different Treatment Conditions^a^

		TSA (μM)			α/β importin inhibitor	
[butyrate]	Control	0.125	0.25	0.5	IV (5 μM)	IM (40 μM)
0	0.38 ± 0.11	0.34 ± 0.13	0.31 ± 0.09	0.24 ± 0.13	0.33 ± 0.16	0.20 ± 0.19
1 mM	0.93 ± 0.07				0.30 ± 0.15	0.32 ± 0.17

a Data represent Manders’ colocalization coefficients of Sin a 1 with DAPI (±SD) obtained from the analysis of 20 CLSM images from 2 independent experiments, using 10 μm Z-stacks; IV: Ivermectin; IM: Importazole.

Moreover, as butyrate is a natural inhibitor of HDAC activities, we carried out experiments to check whether HDAC inhibition could be involved in the nuclear translocation of Sin a 1. For these experiments, we replaced butyrate with a different HDAC inhibitor, trichostatin-A (TSA), and incubated Caco-2 cells with Sin a 1-AF^488^ in the absence or presence of TSA at different concentrations (0.125, 0.25, and 0.5 μM) for 24 h. The analysis of CLSM images (Figure 5) revealed that Sin a 1 remained in the cytoplasm in the presence of TSA regardless of TSA concentration (Manders’ coefficients ranging from 0.24 to 0.34; Table 2). Considering that TSA is a more potent inhibitor of HDAC activities than butyrate, it can be concluded that nuclear translocation of Sin a 1 is independent of HDAC inhibition.

**Figure 5:**
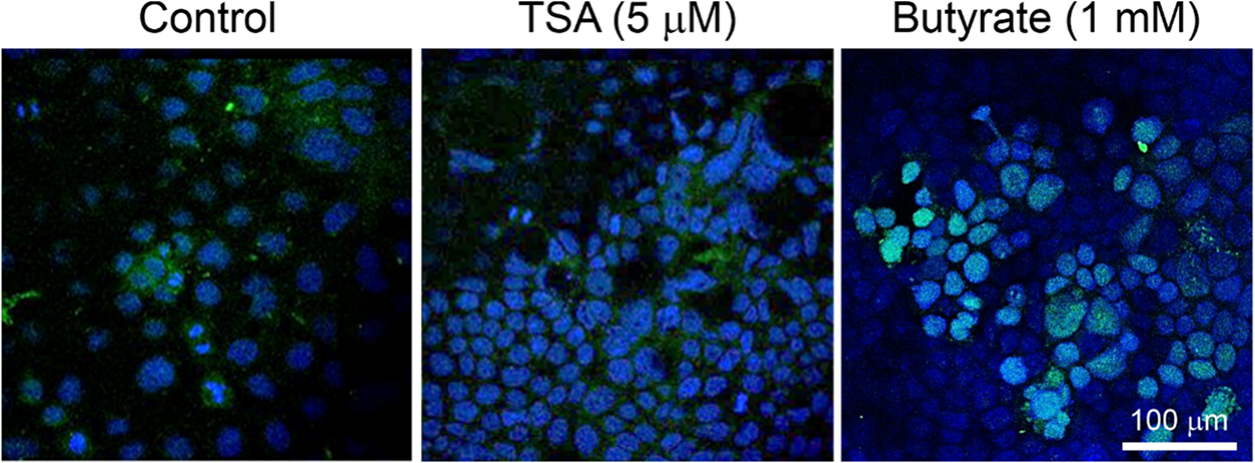
Effect of HDAC inhibition on the nuclear translocation of Sin a 1. Caco-2 cells were incubated for 24 h in the absence or presence of HDAC inhibitors, 1 mM butyrate or 0.5 μM TSA, followed by an additional 24 h of coincubation with 40 μg/mL Sin a 1-AF^488^ (green) in the presence of the HDAC inhibitors. Nuclei were stained with DAPI (blue).

### 3.3 Effect of Sin a 1 on NF-κB Expression

In a first approach to unravel the possible relevance of Sin a 1 nuclear translocation in the presence of butyrate, we checked by qPCR whether Sin a 1 was able to alter NFKB1 (p105/50) transcriptional levels, and if butyrate-induced nuclear translocation modified Sin a 1 potential transcriptional effects. Figure 6 shows that incubation of Caco-2 cells with Sin a 1 induced a highly significant 2.5-fold increase in NF-κB expression, whereas incubation with butyrate alone induced only a 1.5-fold increase. Interestingly, coincubation of cells with butyrate and Sin a 1, under conditions that induced nuclear translocation, significantly decreased the transcriptional effects of Sin a 1 on NF-κB expression to levels comparable to those induced by butyrate alone. This observation suggests that nuclear translocation of Sin a 1 significantly reduces the transcriptional effects of the allergen on NF-κB expression.

**Figure 6:**
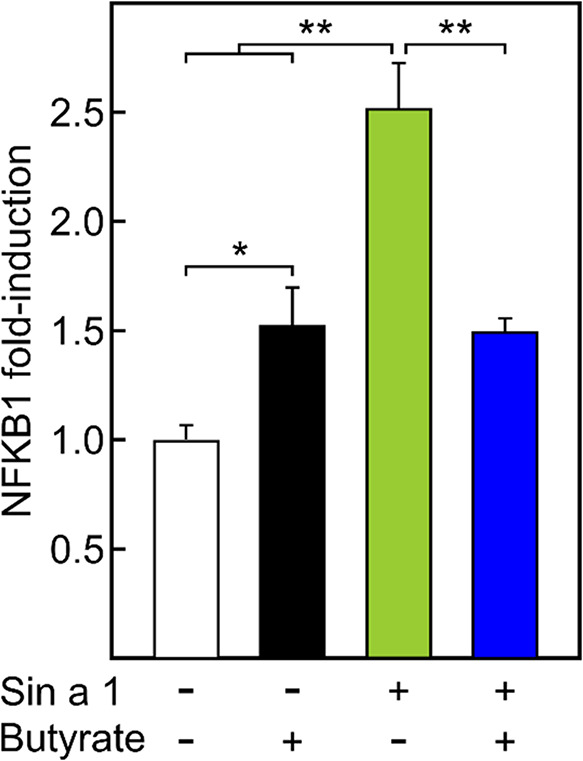
qRT-PCR analysis of NFKB1 expression. Caco-2 cells were preincubated in the absence or presence of 1 mM butyrate for 24 h, followed by an additional 24 h in the presence or absence of Sin a 1. Duplicate independent incubations were carried out with quadruplicate samples. Data correspond to mean fold-change (2^–ΔΔCt^) values ± SD (*) *p* < 0.05; (**) *p* < 0.001.

### 3.4 SCFAs Induce Changes in the Secondary Structure of Sin a 1

To analyze if Sin a 1 nuclear translocation was a consequence of structural alterations in the allergen induced by butyrate, we obtained circular dichroism spectra in the absence and presence of butyrate at different Sin a 1:butyrate molar ratios (1:1, 1:10, and 1:50). Since the translocation is butyrate-specific, we also analyzed the effect of propionate and acetate at the same molar ratios on the secondary structure of the allergen for comparison. Sin a 1 in the absence of SCFAs shows a substantial α-helical content, as evidenced by the two characteristic minima at 208 and 222 nm (Figure 7). According to the secondary structure prediction algorithms (CDNN and CCA), Sin a 1 preparation shows approximately 45% α-helix. Interaction with butyrate induces changes in the CD spectrum characterized by increased negative ellipticity that saturates at a 1:10 molar ratio and corresponds to an increase in α-helix content up to 63–65% (Figure 7A). Since acetate and propionate induce conformational changes equivalent to those observed for butyrate, this change in secondary structure cannot fully account for the activation of the nuclear translocation of Sin a 1 (Figure 7B).

**Figure 7:**
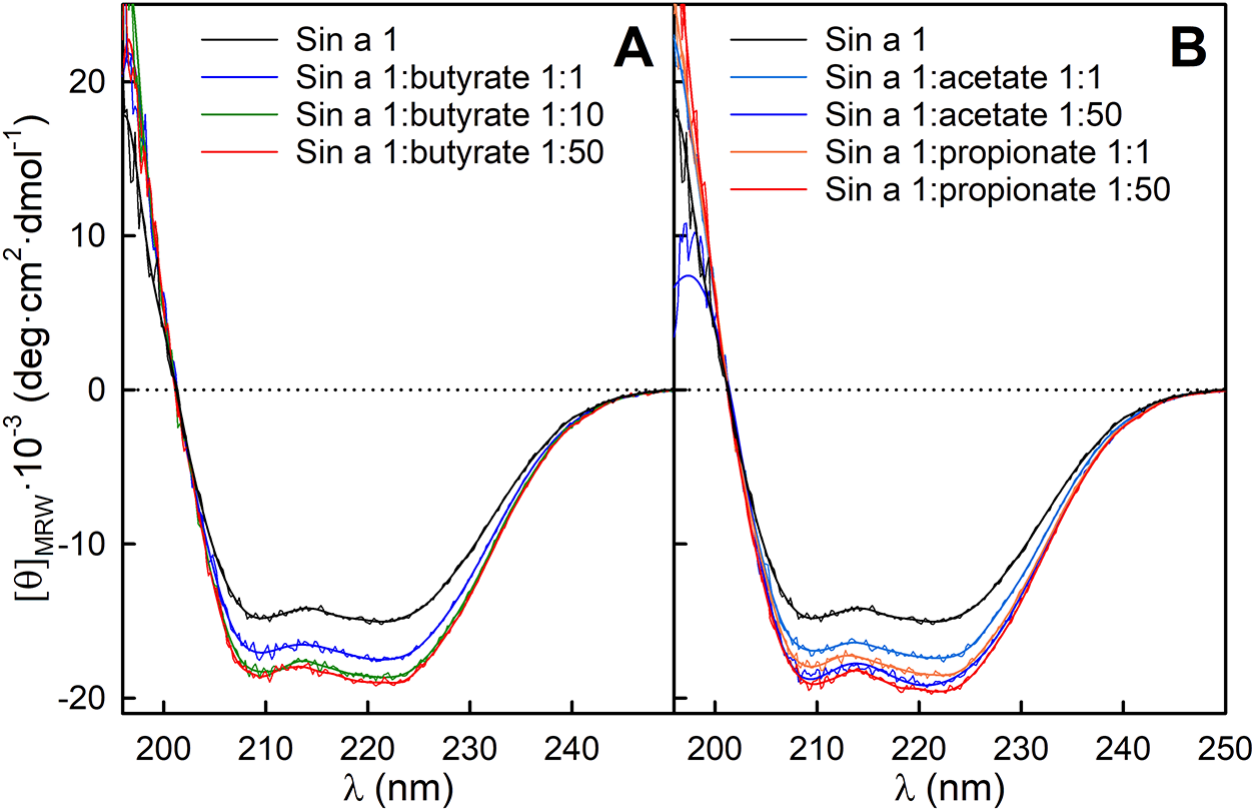
Far-UV circular dichroism (CD) spectra of Sin a 1. Far-UV CD spectra of Sin a 1 (21 μM) recorded in the absence or presence of butyrate (A), acetate, or propionate (B) at the indicated molar ratios. Spectra were recorded in 10 mM HEPES, 0.1 M NaCl, pH 6.5 at 20 °C, and each spectrum represents the average of eight scans after subtraction of the corresponding controls without protein.

### 3.5 Butyrate Specifically Alters the Environment of the Single Tryptophan Residue in Sin a 1

To further investigate the differential effect of butyrate in Sin a 1, we performed a fluorescence emission analysis of the allergen in the presence of the mentioned SCFAs to verify potential conformational changes at the tertiary structure level. In the absence of SCFAs, Sin a 1 spectrum displays a Trp (W^26^) emission maximum at 335 nm, corresponding to a partially buried residue in a hydrophobic environment (Figure 8), while Tyr (Y^117^) emission maximum appears at 304 nm (data not shown), consistent with a buried residue. Sequential addition of butyrate, from 1:0.5 to 1:50 (Sin a 1:butyrate) molar ratios (7 μM Sin a 1), induced a blue shift in W^26^ fluorescence emission, from 335 to 329 nm, in parallel with an increase in the quantum yield, indicating that this residue becomes more deeply buried within the protein core and is less accessible to the solvent (Figure 8A, inset). On the other hand, almost no variation was observed in Y^117^ emission (data not shown). More interestingly, no significant changes in W^26^ or Y^117^ fluorescence emission were induced with the addition of acetate or propionate at the same molar ratios (Figure 8B,C). These results suggest that butyrate induces a conformational change in the environment of W^26^ (located in the light chain) that is specific to this SCFA and could be involved in the translocation of Sin a 1 to the nucleus.

**Figure 8:**
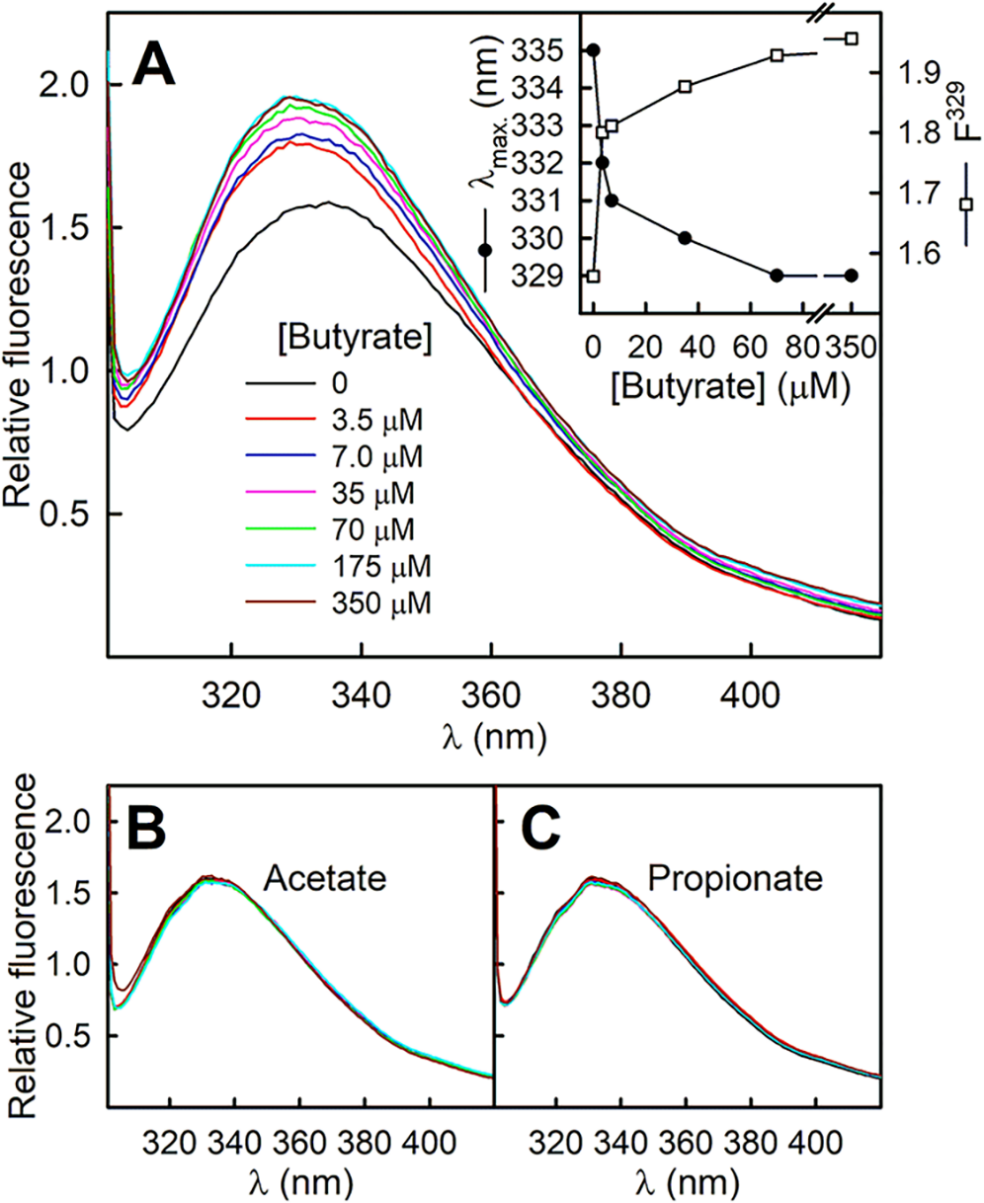
Fluorescence emission spectra of the single Trp residue of Sin a 1 in the presence of SCFAs. Normalized emission spectra of Sin a 1 (7 μM) at an excitation wavelength of 295 nm with increasing concentrations of butyrate (A), acetate (B), or propionate (C) (ranging from 1:0.5 to 1:50 molar ratios, Sin a 1:SCFA). Spectra were recorded at 20 °C in 10 mM HEPES, 0.1 M NaCl, pH 6.5. Only butyrate (A) altered the fluorescence emission spectra in a concentration-dependent manner. The inset shows the blue shift in the position of the emission maximum (black circles) as well as the increase in the quantum yield of W^26^ emission at 329 nm (empty squares).

### 3.6 Prediction of a Potential NLS in Sin a 1

We carried out various *in silico* analyses of the interaction of Sin a 1 with SCFAs, trying to unravel the molecular mechanism involved in nuclear translocation. First, as nuclear internalization via importins requires the exposure of a nuclear localization signal (NLS), we used different online tools to identify potential NLSs. The *NUCDISC* NLS prediction tool (PSORTII server; https://psort.hgc.jp/form2.html) showed that Sin a 1 presents a potential SV40 large T antigen-like NLS on the light chain, localized in the first α-helix, with the sequence: PKCRKEF (pat7 or nuc2 type: P followed within 3 residues by a basic segment containing 3 out of 4 K/R residues). This potential NLS was also confirmed by the INSP server,^53^ although with a shorter sequence (CRKEF). In addition, the nuclear localization of Sin a 1 was predicted by the *k*-nearest neighbors’ classifier algorithm (*k*-NN), yielding a 60.9% probability of nuclear localization,^54^ as well as with Reinhardt’s method for Cytoplasmic/Nuclear discrimination (NNCN score of 94.1).^55^ Thus, it is probable that the NLS in Sin a 1 may be masked and is only exposed under specific conditions, following butyrate interaction, allowing the action of α/β-importins. When we analyzed Pis v 1, Ana o 3 and Pru p 3 sequences, predictions failed to identify any potential NLSs.

### 3.7 Sin a 1 Structure Modeling

In the absence of an experimental Sin a 1 3D-structure, we first performed a homology modeling of Sin a 1. We employed the Swiss-Model server^47^ with the NMR structure of rproBnIb, a 2S albumin from rapeseed (PDB ID: 1SM7),^25^ as a template. We subsequently eliminated residues 40 to 54, which belong to the propeptide of Sin a 1. Additionally, we used the AlphaFold server (AlphaFold 3)^48^ to obtain the model structure using the individual sequences of the light and heavy chains instead of the complete sequence of the precursor. While the overall structure predicted by the two methods was similar, the main differences are in the length of the second and third α-helices from the heavy chain, which are much longer in the AlphaFold 3 model (Figure 9A,B). Interestingly, the Swiss-Model predicts a 41% of α-helical regions, which is in accordance with the calculations based on the CD spectrum in the absence of butyrate. On the other hand, the AlphaFold 3 model shows 63% α-helical content, in accordance with the CD spectra of Sin a 1 in the presence of butyrate, acetate, or propionate.

**Figure 9:**
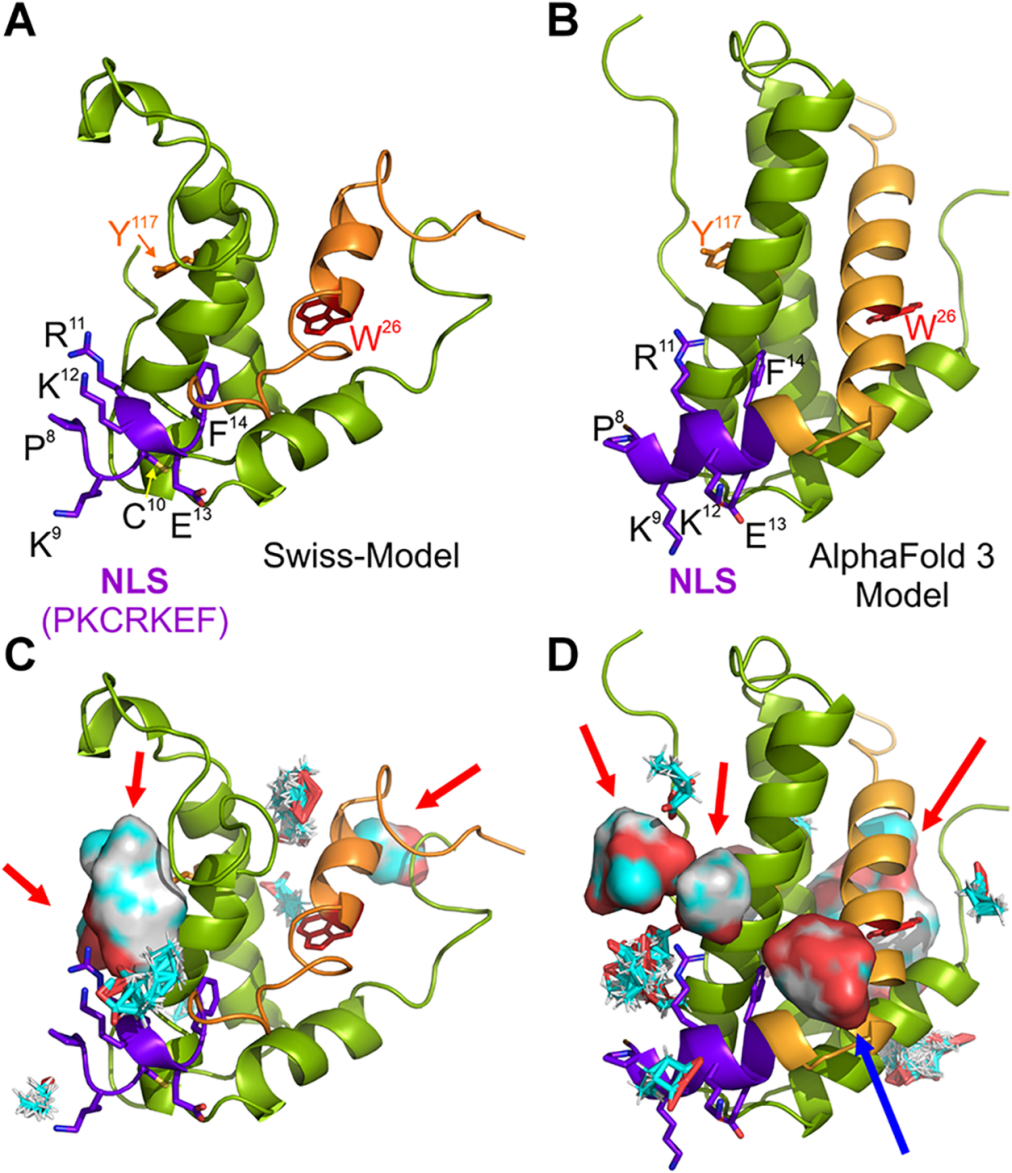
Three-dimensional structural models and SwissDock prediction of butyrate binding sites in Sin a 1. Two different models were obtained based on the amino acid sequence of the allergen. The Swiss-Model server (A) used the structure of rproBnIb, a recombinant 2S albumin from rapeseed, as a template (PDB ID: 1SM7), and predicts approximately 41% α-helical content. On the other hand, the AlphaFold 3 model (B) predicts a higher α-helical content (63%). The potential NLS is marked in magenta, the rest of light chain in orange, and the heavy chain in green. The positions of the single Trp (W^26^, red) and Tyr (Y^117^, orange) residues are indicated. Predicted butyrate binding sites obtained using the SwissDock server are shown, using the Swiss-Model (C) and AlphaFold 3 (D) structures as targets. Butyrate-specific binding clusters are represented as surfaces (red arrows; blue arrow indicates a cluster only predicted in the AlphaFold 3 model), whereas binding sites shared with acetate and propionate are represented as sticks.

### 3.8 SCFA Docking on Sin a 1

We have used the SwissDock tool^49,50^ to map potential SCFA interaction sites on the predicted structural models for Sin a 1 (Figure 9C,D). Butyrate presents distinct binding clusters (displayed as surface representations and marked with arrows), in contrast to acetate and propionate, which showed a higher variability in their potential interaction regions. The side chains of some residues in the potential NLS (mainly R^11^ in both models) are close to one of the butyrate-specific binding regions, likely forming electrostatic interactions with the carboxylic head of butyrate (as predicted by the docking). Acetate and propionate share predicted interaction sites in Sin a 1, some of which overlap with butyrate (represented as sticks in Figure 9), but differ from the butyrate-specific clusters. These results are consistent with the reported conformational changes observed by circular dichroism and W^26^ fluorescence emission. The binding of SCFAs to the shared interaction sites could be responsible for secondary structure changes, whereas the specific binding of butyrate induces burial of W^26^ within the Sin a 1 structure, and may expose the NLS, allowing the interaction with α/β-importins and nuclear translocation. Interestingly, in the AlphaFold 3 model, in which the α-helical content is equivalent to that predicted from the CD spectra of SCFA-bound Sin a 1, an additional butyrate-specific binding site is predicted between helices 1 and 2 of the light chain (blue arrow in Figure 8) and could contribute to stabilizing the conformational change that unmasks the NLS in helix 1.

## 4 Discussion

As detailed in prior studies by our group and others, immature or damaged epithelia exhibit a heightened response to allergens in comparison to fully differentiated and established epithelia.^3,56^ As described in the literature, butyrate is a potent immunomodulatory element of gut epithelia Th2-like responses, promoting tolerance toward new allergens,^18,19,31^ although little is known regarding the molecular mechanisms underlying these effects. Therefore, we decided to analyze the molecular mechanisms by which butyrate may exert these beneficial effects on a cell model consisting of nondifferentiated Caco-2 cells (to resemble an immature or damaged epithelium) treated with Sin a 1, the major allergen from yellow mustard. This model is particularly well-suited for studying plant allergens due to its high thermal stability and resistance to both gastric and intestinal digestion.^28,57^

Using CLSM, we ruled out the presence of the allergen in lysosomes or multilamellar vesicles, as no colocalization of the labeled protein was identified with the mentioned organelles. However, Sin a 1 colocalizes with 14-3-3ζ protein (Manders’ coefficient 0.93), which is noteworthy given the role of this family of proteins as master regulators of intracellular signaling, proteasome regulation^58^ and, most importantly, the regulation of key effectors of the immune response.^59^

Our findings, which were unexpected, revealed that butyrate at a concentration of 1 mM, within the typical range found in the small intestine,^3,8,60^ induces nuclear translocation of Sin a 1-AF^488^ in Caco-2 cells. Colocalization analysis from microscopy images indicated a 93% nuclear localization of Sin a 1 when treated with butyrate, whereas the allergen remained predominantly cytoplasmic under control conditions without butyrate. Moreover, the translocation appears to be specific to this 2S albumin from mustard seeds, as we were unable to reproduce this effect with other 2S albumins from pistachio (Pis v 1) or cashew nuts (Ara o 3) or even with unrelated food allergens such as Pru p 3 from peach skin. Furthermore, we separated the cytoplasm and nucleus of the cells after treatment with Sin a 1-PB and sodium butyrate, and then quantified the fluorescence of each extract. These results strongly suggest that nuclear localization was not an artifact due to the fluorescent probe, as translocation was observed using two different probes.

Considering that butyrate is an inhibitor of HDAC activity,^6,15^ we wanted to check whether nuclear localization could be a consequence of this process. However, we could exclude this possibility, as TSA, a more potent HDAC inhibitor than butyrate,^4,61^ did not induce nuclear translocation of Sin a 1. On the other hand, we have found that nuclear import of Sin a 1 requires the interaction with α/β-importins, as this translocation is blocked by the coincubation with importin inhibitors, importazole and ivermectin, in the presence of butyrate.

Multiple studies have highlighted the multifaceted functions of SCFAs, ranging from immune regulation to metabolism in various tissues and organs. Intestinal microbiota are responsible for the production of SCFAs, with acetate being the most abundant, followed by propionate and butyrate, among other less abundant SCFAs. However, neither acetate nor propionate induced nuclear translocation of Sin a 1, even at concentrations up to 10-fold higher than that used with butyrate.

Does nuclear translocation of Sin a 1 have any effect on the allergenicity of the protein or on the effects of butyrate on Caco-2 cells? We have observed that incubation of Caco-2 cells with Sin a 1 induces a significant increase in the NFKB1 transcription and that this effect is highly reduced when nuclear translocation of Sin a 1 takes place in the presence of butyrate, with expression levels similar to those reached after incubation with butyrate alone.

It was unexpected that treatment with butyrate alone induced transcriptional activation of NFKB1, given that butyrate has been reported to inhibit NF-κB activation and nuclear translocation, thereby modulating the inflammatory response.^20^ However, it is important to note that NFKB1 encodes the precursor protein p105, which undergoes partial proteasomal processing to generate p50, a subunit that forms the active p65/p50 heterodimer. In this context, p105 can also function as an atypical IκB due to the presence of ankyrin-like repeats,^62,63^ and butyrate has been shown to reduce cellular proteasome activity.^64^ This could explain why the butyrate-induced increase in p105 expression does not translate into activation of the NF-κB pathway. In contrast, the allergen in the absence of butyrate induces p105 transcription, and, in the absence of proteasome inhibition, this may lead to increased p50 levels and enhanced formation of the active NF-κB complex. This effect was markedly reduced upon coincubation of Sin a 1 with butyrate.

Although it is tempting to speculate that nuclear translocation accounts for the loss of the Sin a 1-mediated effect on NFKB1 expression, further studies are required to elucidate, on the one hand, how Sin a 1 triggers NFKB1 transcriptional activation and, on the other, how this distinct change in the allergen’s subcellular localization in the presence of butyrate influences this process.

Butyrate and Sin a 1 interact directly with each other *in vitro*, as a significant change in the secondary structure of Sin a 1 is found by CD spectroscopy in the presence of this SCFA. Secondary structure prediction of Sin a 1 based on the CD spectra yields approximately 45% α-helical content, which is very similar to that predicted in the Swiss-Model 3D-structure (41%). On the other hand, after saturation with butyrate, the percentage of α-helix significantly increases to 63–65%, which is more consistent with the 3D model predicted by AlphaFold 3. This variation in α-helical content could potentially influence the targeting of Sin a 1 to the nucleus. However, when we analyzed the effect of acetate and propionate on the secondary structure of the allergen, the changes in the CD spectra were quite identical to those observed with butyrate. A thorough analysis of the 3D models generated in this study reveals a main difference between them, specifically in the loop between helices 2 and 3 of the heavy chain. This loop, as predicted by the Swiss-Model structure, is substantially larger (from A^90^ to H^111^) than that predicted by the AlphaFold 3 model, which includes most of these residues in the mentioned α-helices, exhibiting a shorter loop between them (from G^100^ to G^106^). It is possible that binding of butyrate to Sin a 1 may induce a localized conformational rearrangement of the mentioned loop, adopting a new conformation that better resembles the structure predicted by AlphaFold 3 algorithm for this region.

Furthermore, we were able to detect a conformational change affecting the environment of the single Trp residue based on fluorescence emission spectra, which was specific for butyrate binding. The Trp residue moves toward a more hydrophobic environment, as its fluorescence emission undergoes a blue shift with an increase in its quantum yield. Given the location of this residue at the end of the second α-helix of the light chain, which is part of Sin a 1 coiled-coil, it is reasonable to propose that a slight rotation of this helix may occur, directing W^26^ toward the inside of the coiled-coil. Interestingly, when we analyzed the solvent accessible surface area (SASA) of W^26^ in the Swiss-Model and AlphaFold 3 predicted structures, we found that the former predicted 14% solvent exposure of this residue, whereas the latter only a 9%, which is consistent with the changes observed in Trp fluorescence emission suggesting that Swiss-Model is more accurate for butyrate-free Sin a 1 and that predicted by AlphaFold 3 for the butyrate-bound structure, as also suggested by the comparison of the α-helical content of both models and the CD data.

Considering these findings, it was essential to identify a correlation between the structural changes of Sin a 1, at both the secondary and tertiary structural levels, the involvement of α/β-importins, and the translocation to the nucleus caused by butyrate. First, we used different nuclear localization sequence prediction tools^53,54^ to detect that Sin a 1 contains a potential NLS in the first α-helix of the light chain, with the sequence: PKCRKEF (residues 8–14 of the light chain). According to both structural models described here for Sin a 1, the α-helix starts at the same residue (P^8^), but with low confidence scores according to Alphafold 3 (50–70 pLDDT; predicted local distance difference test) which suggests that this helix, where the potential NLS is located, may be less stable than those which form the coiled-coil (70–90 pLDDT confidence) (Figure S3). This suggests that it may be more sensitive to structural changes that occur after butyrate binding, with the potential unmasking of the NLS.

To identify potential interaction sites between Sin a 1 and the three SCFAs, we conducted docking predictions using the 3D models of the allergen (Swiss-Model, based on the pronapin NMR structure, and AlphaFold 3 model) and the SwissDock server, which predicts binding sites based on the most favorable ΔG interactions. Figure 9 shows three distinct interaction clusters for butyrate in the Swiss-Model structure and four in the AlphaFold 3 model (represented as surfaces). Acetate and propionate showed broader predicted interaction patterns with the allergen, sharing some of the interaction clusters with butyrate. Secondary structure changes detected by CD spectroscopy after interaction with the three SCFAs, involving a significant increase in the overall α-helical content (matching the AlphaFold 3 predicted structure) could be associated with the common binding sites shared by the three SCFAs. Conversely, the specific butyrate binding clusters may promote the movement of the single Trp residue toward a more hydrophobic environment, forcing a conformational rearrangement of the light chain, and exposing the NLS. This would allow interaction with α/β-importins, which mediate the transport of Sin a 1 through the nuclear pore complex. The cargo is then released in the nucleus following Ran-GTP-dependent dissociation from importin β.

Only another 2S albumin from *Glycine max* (soybean; Gly m 8 allergen) has been described to localize in the nucleus of mammalian cells, although these reports mentioned only its light chain, named lunasin.^65,66^ The structure of Gly m 8 is similar to that of Sin a 1, with two polypeptide chains linked by disulfide bonds, and presents a potential nonstandard bipartite NLS according to cNLS mapper,^67^ although with a low score. It is also located in the first α-helix of the light chain (residues 7–30; QDSCRKQLQGVNLTPCEKHIMEK) in an equivalent position to Sin a 1 predicted NLS, but within a more stable α-helix, as predicted by AlphaFold 3 for lunasin (90 > plDDT > 70) (Figure S3). Lunasin has been shown to translocate to the nucleus without requiring an inducer (as butyrate does with Sin a 1), which is in accordance with the more stable α-helix containing the predicted NLS. In any case, it is yet unclear how lunasin enters the nucleus, as it has also been suggested that the interaction with integrins or its association with nuclear basic proteins via its polyaspartic acid C-terminal tail may be involved in its nuclear translocation.

In summary, we present a novel finding: the butyrate-dependent nuclear translocation of a plant allergen, Sin a 1, occurring in mammalian intestinal cells. This translocation occurs via α/β-importins and is not influenced by the inhibitory effect of butyrate on HDACs. Although other SCFAs, such as acetate and propionate, do bind to Sin a 1 and induce comparable changes in the secondary structure of the allergen (more than 20% increase in α-helical content), they do not trigger nuclear translocation. Docking predictions suggest that Sin a 1 presents butyrate-specific binding sites, apart from those common to all SCFAs. These interactions slightly alter the conformation of the light chain. This alteration, which modifies the single Trp residue environment located at the end of this chain, may potentially unmask the NLS present in the first α-helix of this subunit. These results demonstrate that, in the context of allergies, butyrate not only acts on cells but also directly interacts with the allergen before its epithelial uptake. The findings of this study offer a novel approach to the study of butyrate-induced tolerance by focusing on direct protein–ligand interactions rather than its direct impact on cells.

## Supplementary Material



## Notes

Materials and Methods: Preparation of delipidated mustard seed extracts; primer sequences employed on qPCR assays (Table S1); colocalization of labeled allergens with DAPI (Table S2); SDS-PAGE and Western blot analysis of purified Sin a 1 from yellow mustard seeds (Figure S1); analysis of the efficacy of the separation of cytoplasmic and nuclear extracts (Figure S2); and AlphaFold 3 prediction of the structures of Sin a 1 and Gly m 8 allergens (Figure S3) (PDF)
