# Supplementary material for: Butyrate-Dependent Conformational Switch Promotes Nuclear Translocation of the Mustard Allergen Sin a 1 in Human Gut Epithelial Cells
Source: J Agric Food Chem. 2026 Jun 11;74(24):19053–65. doi: 10.1021/acs.jafc.6c01248 (PMC13307368; doi:10.1021/acs.jafc.6c01248)
Supplement: Supplementary file 1 [file jf6c01248_si_001.pdf]

Supporting Information

A butyrate-dependent conformational switch promotes nuclear translocation of the mustard allergen Sin a 1 in human gut epithelial cells

*Rubén G. Gordo, Jorge Parrón-Ballesteros, Nieves Olmo, Mayte Villalba, Eva Batanero and Javier Turnay**

Department of Biochemistry and Molecular Biology, Faculty of Chemistry, Complutense University of Madrid, 28040-Madrid, Spain

(*) Corresponding author: Javier Turnay: turnay@ucm.es; phone: +34913944148

Materials and Methods

Preparation of delipidated mustard seed extracts

Mustard seeds were ground in the presence of liquid nitrogen and exhaustively delipidated with cold acetone. The organic phase was subsequently discarded, and the resulting flour was air-dried overnight to allow complete acetone evaporation. Protein extraction was performed by homogenization in 100 mM citrate buffer (pH 3.5) supplemented with a protease inhibitor cocktail (Thermo Scientific, Alcobendas, Spain). The extract was filtered through Miracloth (Millipore, Madrid, Spain), and the buffer was exchanged to 20 mM ammonium bicarbonate (pH 6.5) using a PD-10 Sephadex G-25M column (Fisher Scientific, Alcobendas, Spain). The resulting preparation was lyophilized and stored at -20 °C until use.

Table S1. Primer sequences employed on qPCR assays.

Gen	Accession number	Protein	Forward Primer	Reverse Primer	Expected size
NFKB1	NM_003998	NF-κB1 (p105/50)	5'-CCTGGAACCACGCCT-3'	5'-CATGTCTCCTTGTGCTAG-3'	114 bp
EF1A1	NM_001402	EF-1α	5'-CTGAACCATCCAGGCCAAAT-3'	5'- GCCGTGTGGCAATCCAAT -3'	59 bp

Table S2. Colocalization of labeled allergens with DAPI.

	**Sin a 1**	**Pis v 1**	**Ana o 3**	**Pru p 3**
Control	0.38 ± 0.11	0.38 ± 0.04	0.26 ± 0.02	0.36 ± 0.08
Butyrate (1 mM)	0.93 ± 0.07	0.35 ± 0.10	0.27 ± 0.08	0.37 ± 0.09

Data represent mean Manders' colocalization coefficients of Alexa Fluor 488-labeled allergens with DAPI (± SD) obtained from the analysis of up to 20 CLSM images from 2 independent experiments, with Z-stacks captured at 10 μm.

FIGURE S1

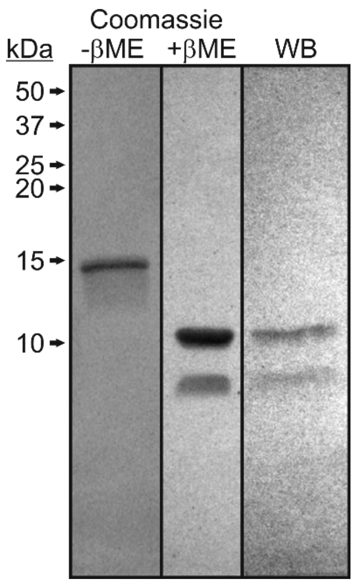

Figure S1. SDS-PAGE and Western blot analysis of purified Sin a 1 from yellow mustard seeds.

Electrophoresis was carried out under non-reducing (-βME) or reducing (+βME) conditions, and gels were stained with Coomassie blue. Western blot (WB) was performed after SDS-PAGE under reducing conditions and using a rabbit polyclonal antibody (kindly provided by Dr. Carlos Pastor-Vargas, Complutense University of Madrid; 1:2,000) followed by an HRP-conjugated goat anti-rabbit (31437X, 1:5,000, Invitrogen). Band were visualized using ECL Western Blotting Substrate (Thermo Scientific), and images were acquired at different exposure times in a ChemiDoc XRS+ system (Bio-Rad).

FIGURE S2

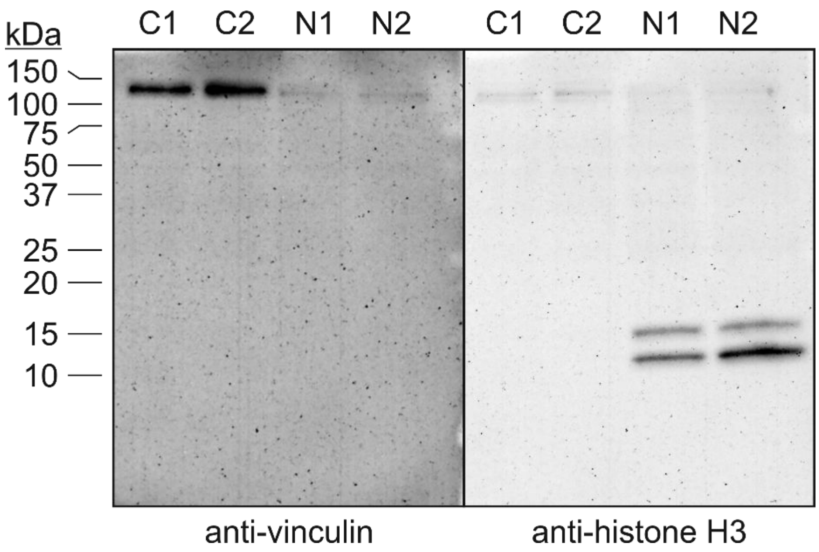

Figure S2. Validation of cytoplasmic and nuclear extract separation from Caco-2 cells.

Nuclear and cytoplasmic extracts from Caco-2 cells under different incubation conditions were obtained using the NE-PER Nuclear and Cytoplasmic Extraction kit (*Thermo Scientific*). Lanes C1 and C2 correspond to cytoplasmic extracts from cells treated with butyrate and Sin a 1/butyrate, respectively, whereas N1 and N2 are the corresponding nuclear extracts. The membrane was first incubated simultaneously with both primary antibodies (mouse monoclonal anti-human vinculin and rabbit polyclonal anti-human histone H3), followed by incubation with HRP-conjugated anti-mouse IgG and developed using ECL. Afterwards, the membrane was stripped and reprobed using HRP-conjugated anti-rabbit IgG and further developed.

FIGURE S3

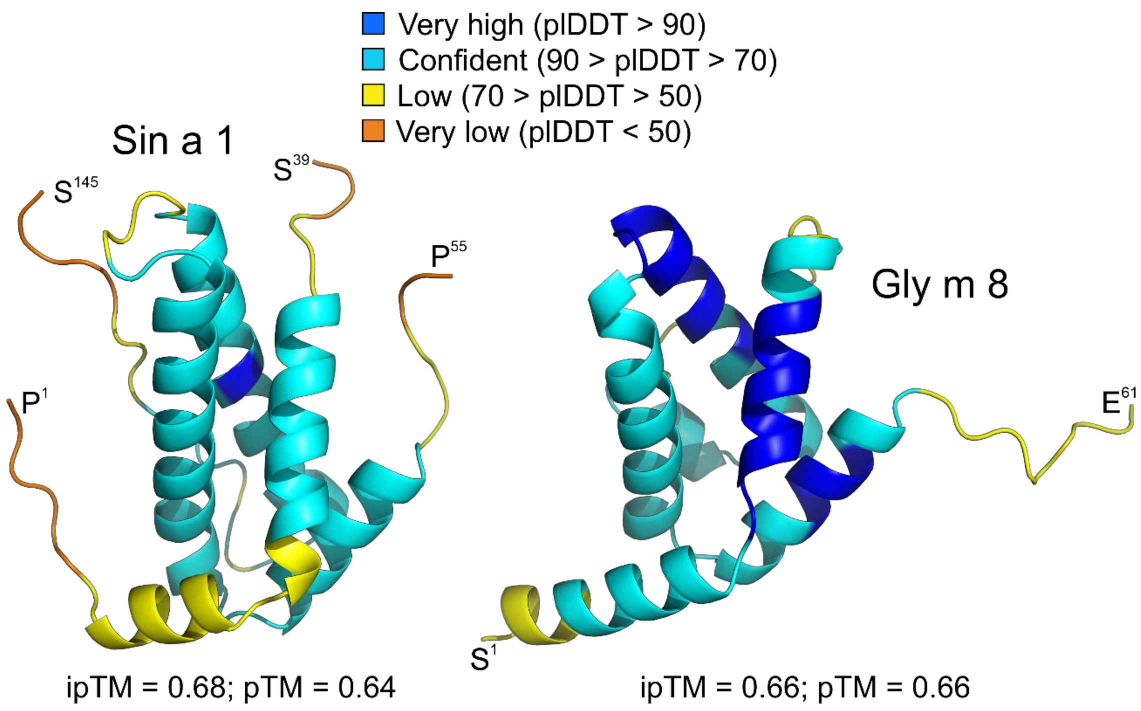

Figure S3. AlphaFold 3 structural predictions of Sin a 1 and Gly m 8 allergens.

Modeling of the 3D structures was obtained at the AlphaFold server using the individual sequences of the light and heavy chains separately. Colors indicate pLDDT values (predicted local distance difference test: a per-atom confidence estimate on a 0-100 scale with higher value indicating greater confidence). pTM (predicted template modeling score) and the interface predicted template modeling score (ipTM) are also included. pTM > 0.5 indicates that the overall predicted fold for the complex might be similar to the true structure. ipTM measures the accuracy of the predicted relative positions of the two chains in the model and must be above 0.6.
